# What leads users to recommend, not just use? Unpacking service quality, labor perceptions, and conscious consumer choice in food delivery apps

**DOI:** 10.3389/fsoc.2025.1666695

**Published:** 2025-10-17

**Authors:** Jorge Serrano-Malebrán, Carlos Molina

**Affiliations:** ^1^Facultad de Ingeniería y Negocios, Escuela de Ingeniería Comercial, Universidad de las Américas, Santiago, Chile; ^2^Facultad de Economía y Administración, Departamento de Administración, Universidad Católica del Norte, Antofagasta, Chile

**Keywords:** delivery apps, service quality, socially conscious consumption, working conditions, intention to recommend, SOBC model

## Abstract

This study examines how perceptions of service quality, working conditions, and socially conscious consumption influence the intention to use and recommend food delivery applications in Chile. Drawing on the Stimulus–Organism–Behavior–Consequence (SOBC) theoretical framework, this study simultaneously analyzed functional factors, namely efficiency, fulfillment, system availability, and privacy, and ethical-social factors, such as perceived working conditions. A quantitative design was employed with a sample of 416 users, evaluating the constructs through validated scales and applying Partial Least Squares Structural Equation Modeling (PLS-SEM). The results indicate that service quality is the strongest predictor of intention to use and positively affects both the intention to recommend and socially conscious consumption. In contrast, working conditions do not directly influence intention to use but positively impact intention to recommend and socially conscious consumption. Socially Conscious consumption, in turn, influences only the intention to recommend and not the intention to use. The findings confirm that, in digital contexts, socially conscious consumers tend to express their values more through recommendations than through purchase decisions. These insights offer relevant implications for academia and practice, suggesting that integrating operational efficiency with fair labor practices can strengthen the sustainability of platform-based business models.

## 1 Introduction

Over the past decade, digital food delivery platforms have undergone significant expansion, with projected revenues reaching USD 1.35 trillion by 2025 (Statista, 2025), This growth has been fueled by the digitalization of consumption and further accelerated by the COVID-19 pandemic (Belanche et al., 2021). These platforms have consolidated their role as key players in the on-demand service economy, transforming consumption patterns and labor regimes across multiple regions worldwide (Goods et al., 2024). Their business models, often reliant on subcontracting and algorithmic control, shift operational risks to workers and reproduce employment schemes marked by instability, digital surveillance, and a lack of social protection (Mendonça and Kougiannou, 2023). In particular, delivery riders face physical insecurity, economic dependency, and limited autonomy, raising ethical concerns about the social sustainability of these platforms (Bonhomme and Muldoon, 2024; Goods et al., 2024). Within this context, a growing body of research has sought to understand how consumers integrate functional and social evaluations when deciding whether to use or recommend such services. The literature suggests that socially conscious consumers are more likely to penalize platforms that fail to ensure fair working conditions (Ali et al., 2023; Belanche et al., 2021). However, functional attributes related to system quality, such as efficiency, delivery fulfillment, ease of use, and privacy, remain the key determinants of usage decisions (Vu et al., 2023; Wang et al., 2024).

Despite these advances, there is still no empirical consensus on how these stimuli are prioritized or integrated into consumer behavior. Some studies argue that socially conscious consumption may act as a mediator or moderator between perceptions and behavior (Ali et al., 2023), while others suggest that its influence can be neutralized by utilitarian incentives such as low prices or urgent consumption needs (Erkmen and Turegun, 2022; Goods et al., 2024). This theoretical ambiguity reveals a critical gap: the interaction between functional and ethical-social stimuli in shaping behavioral responses is not yet well understood, nor is the differential impact of these factors on intention to use versus intention to recommend.

To address this gap, this study draws on the Stimulus–Organism–Behavior–Consequence (SOBC) theory developed by Davis and Luthans (1980), which has previously been applied in studies on food delivery and online shopping behavior (Awal et al., 2024; Chakraborty, 2025; Talwar et al., 2021). This model proposes that stimuli (S), defined as environmental and interactive events, influence an individual’s cognitive processing (organism – O), which, in turn, leads to observable behavioral responses (behavior – B), ultimately resulting in social or relational consequences (consequence – C) (Chakraborty, 2025; Talwar et al., 2021). Unlike frameworks that stop behavioral responses, the SOBC model captures downstream effects, such as service recommendation, reputational reinforcement, or symbolic punishment of certain platforms. In this study, the stimuli are represented by perceived service quality and working conditions, socially conscious consumption acts as the evaluative organism that processes these stimuli, intention to use reflects immediate behavior, and intention to recommend constitutes the consequence emerging from that behavior.

Based on this conceptualization, the general objective of this study was to analyze how perceptions of service quality, working conditions, and socially conscious consumption influence the intention to use and recommend food delivery applications. The specific objectives were: (1) to assess the effect of service quality, through the dimensions of efficiency, fulfillment, system availability, and privacy, on intention to use and recommend; (2) to analyze how socially conscious consumption shapes the relationship between stimuli and behavior; and (3) to examine the impact of perceived working conditions on intention to use and recommend. This approach seeks to integrate utilitarian and social motivations and to understand the behavioral consequences of these evaluations within the context of the platform economy.

## 2 Literature review

### 2.1 The Stimulus–Organism–Behavior–Consequence (SOBC) model

The Stimulus–Organism–Behavior–Consequence (SOBC) model, proposed by Davis and Luthans (1980), constitutes an evolution of Mehrabian and Russell’s classic S–O–R framework (Mehrabian and Russell, 1974), extending its scope by incorporating an explicit behavioral stage between cognitive processing and observable consequences. This model has been widely applied to explain a range of individual behaviors, particularly in organizational and consumer contexts (Chakraborty, 2025; Talwar et al., 2021). The SOBC model posits that stimuli (S), understood as interactive environmental events, influence the organism (O), that is, an individual’s cognitive processes. This internal evaluation leads to an observable behavior (B), which in turn generates a consequence (C) derived from the action (Davis and Luthans, 1980). This sequence has been validated in diverse contexts, including ethical consumption behavior (Dhir et al., 2020), intention to adopt smart technologies (Saheb et al., 2022), and consumer responses to digital overload (Whelan et al., 2019).

In the context of digital food delivery platforms, the SOBC model provides a robust framework for analyzing how consumers simultaneously process functional and ethical-social stimuli. In this study, these are conceptualized as service quality and perceived working conditions. These dimensions operate as external influences that activate internal evaluative processes within the organism, represented by socially conscious consumption (SCC), a cognitive disposition to integrate ethical and social values into decision-making. This evaluation, in turn, leads to a specific behavior: the intention to use the application, which ultimately results in a socially observable consequence: intention to recommend the service to others.

From a functional perspective, service quality has been extensively studied as a key driver of consumer behavior in digital environments. The E-S-QUAL model, which includes the dimensions of efficiency, fulfillment, system availability, and privacy, has proven effective in explaining satisfaction, loyalty, and intention to use delivery service apps (Parasuraman et al., 2005; Wang et al., 2024). For example, attributes such as efficient navigation and loading times, timely and error-free deliveries, and secure transactions have been found to contribute directly to repeated usage and service recommendations (Belanche et al., 2021; Vu et al., 2023).

In parallel, the perceived working conditions of delivery riders represent a salient ethical-social stimulus. Recent studies show that consumers, particularly those with high levels of social consciousness, tend to penalize platforms associated with precarious labor practices, such as a lack of social protection or limited worker autonomy (Goods et al., 2024; Kim et al., 2023). These stimuli can activate critical cognitive processes (organisms) that shape consumer behavior.

Socially conscious consumption, as a central cognitive component of the model, reflects consumers’ critical evaluations of environmental stimuli. Prior research has shown that this variable influences both usage behavior and subsequent social actions such as recommending or criticizing the service (Ali et al., 2023; Belanche et al., 2021). However, this consciousness may be more strongly expressed through symbolic or relational actions, such as public recommendations or disapproval, than through immediate behavioral decisions, such as using or avoiding a platform (Vu et al., 2023).

Finally, intention to recommend is understood as a downstream behavioral consequence that socially validates or sanctions the consumption experience. Several studies have demonstrated that consumers who perceive a service to be efficient, ethical, and reliable often act as informal promoters, thereby amplifying a platform’s reputational value in digital environments (Fan et al., 2022; Kim et al., 2023). Taken together, the SOBC model offers an integrated perspective to capture the influence of environmental factors, cognitive evaluations, resulting behaviors, and social consequences. This framework is especially useful for understanding how functional assessments and perceptions of labor justice interact within the platform economy.

Within the Stimulus–Organism–Behavior–Consequence (SOBC) framework, we conceptualize intention to use as the behavioral stage (B) and intention to recommend as the consequence stage (C). This mapping follows prior SOBC applications in consumer contexts where a proximal behavioral response precedes a downstream, socially visible consequence (Awal et al., 2024; Talwar et al., 2021). In our case, usage reflects an immediate behavioral act, whereas recommendation represents a relational validation, consistent with SOBC’s broader scope from environmental stimulus to reputational consequence.

Although the canonical SOBC sequence is S → O → B → C, digital service environments often display direct “shortcut” effects from stimuli or organism to consequences. For example, consumers may recommend a platform based on efficiency, fulfillment, or fairness without altering their own usage behavior (Fan et al., 2022; Vu et al., 2023). Retaining these links is theoretically justified, as prior work on service quality shows that E-S-QUAL dimensions can drive both loyalty intentions and advocacy directly (Parasuraman et al., 2005; Wang et al., 2024). In the gig-economy setting, perceptions of labor practices also operate as salient stimuli that can influence use and recommendation differentially, while socially conscious consumption functions as a cognitive–affective filter of these evaluations (Belanche et al., 2021; Goods et al., 2024).

### 2.2 Service quality

Multiple studies have demonstrated that consumers evaluate service quality based on their operational experience with the platform, which directly influences their cognitive processes (organism), shapes their behavioral intentions (application usage), and eventually leads to social consequences, such as service recommendation (Belanche et al., 2021; Erkmen and Turegun, 2022). One of the most widely used frameworks for operationalizing this construct in digital environments is the E-S-QUAL model, proposed by Parasuraman et al. (2005), which has been extensively adapted to the context of delivery applications. This model conceptualizes electronic service quality as a multidimensional construct comprising efficiency, fulfillment, system availability, and privacy. Each of these dimensions has significant effects on satisfaction, intention to use, and loyalty in digital settings (Fan et al., 2022; Vu et al., 2023).

Efficiency, defined as the ease and speed with which users can navigate the application and complete transactions, has been identified as a key dimension that influences user trust (Parasuraman et al., 2005). Fulfillment refers to the platform’s ability to deliver accurately on promises regarding time, product, and condition and is consistently cited as one of the most valued attributes by consumers (Kim et al., 2023). System availability, which relates to technical reliability and the absence of errors or crashes, is directly associated with the continued usage of the service (Vu et al., 2023). Finally, privacy, though less visible to users, is critical for fostering a perception of security in digital environments (Goods et al., 2024).

Beyond its direct influence on consumer behavior, service quality can also act as a cognitive trigger that facilitates socially conscious consumption as it constitutes the initial interpretive framework through which users assess the legitimacy and values of a platform (Ali et al., 2023). When the service is perceived as efficient, reliable, and aligned with user expectations, consumers may be more inclined to reflect critically on other dimensions such as labor conditions or the company’s social responsibility commitments (Erkmen and Turegun, 2022; Vu et al., 2023). In line with the reviewed literature and following the SOBC model, the following hypotheses are proposed:

*H1:* Perceived service quality positively influences socially conscious consumption.

*H2:* Perceived service quality positively influences the intention to use.

*H3:* Perceived service quality positively influences the intention to recommend.

### 2.3 Working conditions

In the context of food delivery platforms, working conditions are often shaped by subcontracting arrangements, absence of social protection, uncertain remuneration, and algorithmic control, which have been widely documented as constituting a precarious work environment (Belanche et al., 2021; Goods et al., 2024). These conditions have gained increasing media and public visibility through reports of workplace accidents, legal disputes, and worker protests, which have raised awareness among certain segments of the consumer public regarding labor justice on these platforms (Belanche et al., 2021; Tsarenko et al., 2019). Empirical studies have shown that negative perceptions of working conditions can directly influence consumer behavioral intentions, particularly among ethically sensitive users who may reduce their intention to recommend the service (Belanche et al., 2021; Kim and Jang, 2019).

Robust evidence also indicates that some consumers are willing to accept certain utilitarian sacrifices, such as longer wait times or modest price premiums, when they perceive that a platform is actively improving the working conditions of delivery workers (Belanche et al., 2021). However, this willingness is not uniform; while socially conscious consumers tend to penalize platforms perceived as unfair, other users prioritize convenience, price, or service speed (Audrain-Pontevia et al., 2013; Goods et al., 2024).

Moreover, many platforms operate under schemes that shift the operational costs to workers. This includes paying for their own vehicles, lacking insurance coverage, facing pressure to accept orders under rating-based systems, and being subject to the algorithmic management of tasks (Jabagi et al., 2019). Such organizational logic creates labor conditions that are intensive, risky, and opaque, and conditions that some consumers perceive as incompatible with their personal values, thereby activating internal evaluative processes.

From the perspective of the SOBC model, these perceptions function as environmental stimuli that shape an individual’s cognition (organism), which, in turn, affects their behavioral intention (service usage) and social consequences (recommendation). In other words, consumers filter these stimuli through their values and beliefs, which may be reflected in decisions that either favor or reject certain platforms (Belanche et al., 2021). Based on this evidence, the following hypotheses are proposed:

*H4:* Perceived working conditions positively influence socially conscious consumption.

*H5:* Perceived working conditions positively influence the intention to use food delivery applications.

*H6:* Perceived working conditions positively influence the intention to recommend food delivery applications.

### 2.4 Socially conscious consumption

Socially conscious consumption reflects consumers’ willingness to consider the social impact of their purchasing decisions, particularly in precarious labor contexts, such as those of food delivery platforms (Belanche et al., 2021). This concern may lead to reduced intention to recommend such services. Recent studies have shown that consumers with high levels of social consciousness are especially sensitive to the working conditions of delivery workers, expressing lower intention to use and intention to recommend platforms perceived as unfair, even when these platforms offer functional benefits such as speed or low prices (Kim et al., 2023). This ethical evaluation not only shapes behavioral intentions but also influences consumers’ overall perception of the service by incorporating normative concerns into their functional judgment (Ali et al., 2023).

The literature has also conceptualized social consciousness as an individual trait that can mediate or moderate the relationship between perceived stimuli, such as service quality or working conditions, and behavioral intentions, such as using or recommending a service (Vu et al., 2023). For example, socially conscious consumers have been found to be more willing to make trade-offs in terms of convenience or price if they perceive that a company treats its workers fairly (Kim et al., 2023). Moreover, they tend to prefer platforms that actively communicate their commitment to labor well-being, even if this entails additional costs (Belanche et al., 2021).

Within the SOBC framework, socially conscious consumption translates environmental stimuli into concrete behavioral intentions and may also influence the nature of the relational consequences that users are willing to assume, such as recommending or actively promoting certain services. Based on this evidence, the following hypotheses are proposed:

*H7:* Socially conscious consumption positively influences intention to use food delivery platforms.

*H8:* Socially conscious consumption positively influences intention to recommend such platforms.

### 2.5 Intention to recommend

In the context of food delivery platforms, the intention to recommend reflects not only customer satisfaction with the service received but also the evaluative judgment that consumers form regarding the functional and social legitimacy of the business model (Belanche et al., 2021). Various studies have shown that recommendation intentions are strongly associated with positive usage experiences in which users perceive operational efficiency and responsible business practices (Yeo et al., 2017). This relationship has been documented in both European and Latin American markets, where recommendation is understood not only as a utilitarian act, but also as a symbolic one: socially sensitive users often express their approval by actively promoting the service, reinforcing their identity as socially conscious consumers (Belanche et al., 2021).

Within the SOBC framework, intention to recommend is conceptualized as the consequence (C) of an observable behavior (B); in this case, intention to use, which is in turn influenced by internal organism processes (cognitive and affective evaluations) and environmental stimuli (Davis and Luthans, 1980; Talwar et al., 2021). Accordingly, consumers who report a high intention to use are more likely to act as informal ambassadors of the service (Vu et al., 2023).

Furthermore, the literature emphasizes that both functional and ethical-social stimuli affect this behavior. In particular, consumers with high levels of social consciousness are more likely to recommend platforms that ensure decent working conditions, even if doing so entails sacrificing functional aspects, such as price or speed (Goods et al., 2024). From this perspective, recommendations serve as a form of moral validation aimed at encouraging more just and sustainable business models. Based on this evidence, we propose the following hypothesis:

*H9:* The intention to use positively influences the intention to recommend food delivery services.

**Figure 1:**
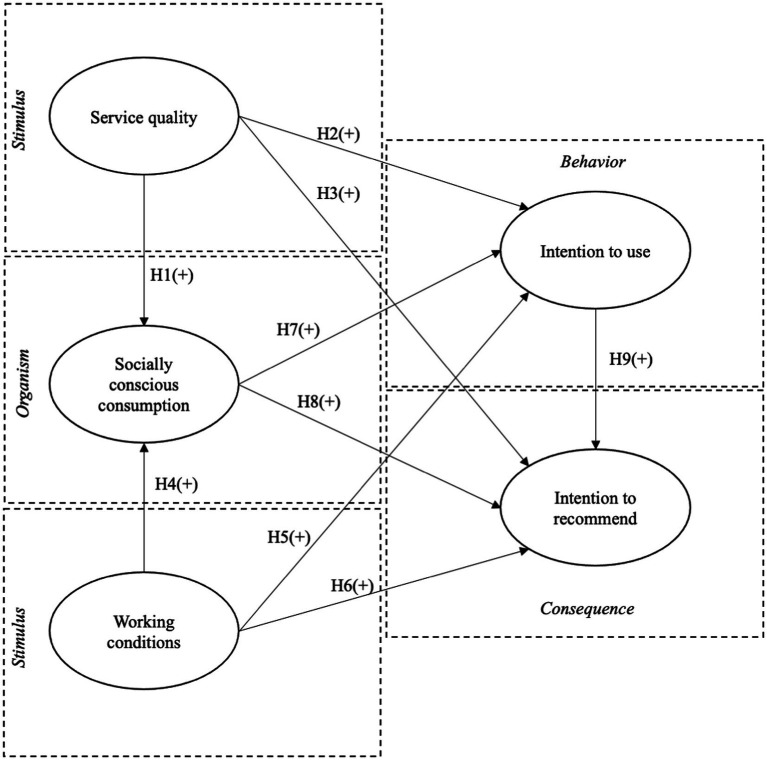
Proposed model.

Figure 1 presents the conceptual model based on the Stimulus–Organism–Behavior–Consequence (S–O–B–C) framework. The model posits that perceived service quality and working conditions act as external stimuli (S) that influence the internal evaluative process of socially conscious consumption (O). This internal state, in turn, is associated with two key outcomes: the intention to use food delivery applications (behavior) and the intention to recommend such services to others (consequence). The model reflects a sequential logic whereby functional and ethical-social perceptions trigger value-based evaluations, which then lead to individual and relational behavioral responses. This structure enables the analysis of how users integrate operational performance and labor-related concerns into their decision-making processes, particularly distinguishing between personal usage and public endorsement of digital platforms.

## 3 Methodology

### 3.1 Sample and pretest

This study employed a quantitative, cross-sectional design to analyze functional and ethical–social factors influencing intentions to use and recommend digital food delivery platforms. Data were collected between October and November 2023 across different regions of Chile via a structured questionnaire administered by professional interviewers. The final sample comprised 416 valid responses obtained through a non-probability intercept design. Participants were recruited through interviewer-administered intercepts at high-traffic public venues (e.g., shopping centers, parks, transit hubs) located across the northern, central, and southern macro-zones of Chile. To enhance coverage and reduce temporal biases, fieldwork was distributed across weekdays and weekends and included both daytime and evening sessions. Eligibility criteria required respondents to be residents of Chile, aged 18 or older, active users of food delivery apps with internet access, and to have placed at least one order in the month prior to the survey. Interviewers applied soft quotas for gender, age, and macro-zone based on official national statistics for digital service users (SUBTEL, 2017) to approximate the demographic distribution of the target population. Regarding the sample profile, 49.3% identified as women, 49.3% as men, and 1.4% as another gender. In terms of age, 50% were 21–30 years old, 18.3% were 31–40, and 14.4% were 18–20. Geographically, 56.5% resided in the central zone, 22.6% in the northern zone, and 20.9% in the southern zone. Most participants were single (75.7%). PedidosYa was the most frequently used application (44%), followed by Uber Eats (7.7%). On average, participants reported spending CLP $10,000–$20,000 per month on app-based orders (45.9%), with debit cards being the most common payment method (42.3%).

### 3.2 Measurement scales

The data collection instrument consisted of a structured questionnaire administered by professional interviewers using a digital form. The first section gathered basic sociodemographic information (e.g., gender, age, educational level, marital status). The second section comprised items designed to measure the variables included in the analytical model: perceived service quality, perceived working conditions, socially conscious consumption, intention to use, and intention to recommend food delivery applications. Perceived service quality was operationalized using the E-S-QUAL model (Parasuraman et al., 2005), which includes four dimensions: efficiency, fulfillment, system availability, and privacy. In line with E-S-QUAL, the Privacy dimension captures users’ perceptions about the protection of personal and financial data. Respondents evaluate whether apps protect their purchase information, safeguard credit card details, and avoid sharing personal data with other parties. These judgments are based on observable cues such as privacy notices, payment security features, or app permissions, rather than direct verification of back-end practices. Perceived working conditions were measured with scales related to internal social responsibility and labor ethics adapted from Öberseder et al. (2013), assessing perceptions of organizational justice and labor rights protection. Socially conscious consumption was evaluated using a scale adapted from Pepper et al. (2010). Intention to use was measured with items based on Belanche et al. (2011), and intention to recommend with items derived from Harrison-Walker (2001). All variables were measured on a 7-point Likert scale (1 = “Strongly disagree” to 7 = “Strongly agree”).

To ensure linguistic and cultural equivalence for the Spanish-speaking context, we followed a forward–back translation procedure (Brislin, 1980). Two bilingual researchers independently translated the original English items into Spanish, and a third researcher—blind to the originals—back-translated the Spanish version into English. Discrepancies were discussed and resolved by consensus to preserve semantic and conceptual equivalence. Prior to full deployment, the instrument underwent a two-stage pretesting process: (i) a pilot with a student subsample to refine wording and ensure semantic clarity, and (ii) a field pretest with 25 respondents from the target population to assess cultural adequacy and comprehension. Minor wording adjustments were incorporated based on feedback before launching the full survey.

### 3.3 Statistical tools

Data analysis was conducted using Partial Least Squares Structural Equation Modeling (PLS-SEM), a statistical technique widely used in social and behavioral sciences because of its ability to model complex relationships among latent variables, control for measurement errors, and test comprehensive theoretical frameworks (Gudergan et al., 2008; Henseler et al., 2016). This approach is particularly suitable for exploratory research or models involving a large number of constructs and indicators, as is the case in this present study. Moreover, PLS-SEM offers flexibility regarding distributional assumptions and sample size requirements, making it especially well suited for applied research in digital marketing and consumer behavior on platform-based services. Following the methodological guidelines of Hair et al. (2022), the analysis was structured in two stages: first, the evaluation of the measurement model to assess the reliability and validity of the constructs, and second, the evaluation of the structural model to test the proposed hypotheses. Both stages were conducted using the SmartPLS 4 software (Ringle et al., 2022).

## 4 Results

### 4.1 Measurement model assessment

Three key criteria of the measurement model were assessed to ensure the psychometric quality of the constructs: internal reliability, convergent validity, and discriminant validity. This evaluation followed established methodological recommendations (Fornell and Larcker, 1981; Henseler et al., 2016; Sarstedt et al., 2017) using the Partial Least Squares Structural Equation Modeling (PLS-SEM) approach. Cronbach’s Alpha (CA) and Composite Reliability (CR) were used to assess the internal consistency of the constructs. As shown in Table 1, all the CA and CR values exceeded the recommended threshold of 0.70, indicating adequate internal reliability for each construct (Hair et al., 2022). Convergent validity was assessed using two indicators: individual item factor loadings and Average Variance Extracted (AVE). All item loadings exceeded the minimum accepted threshold of 0.70, and the AVE values were greater than 0.50, thus supporting the convergent validity of the constructs (Chin, 1998; Fornell and Larcker, 1981). System Availability, measured with two items after indicator refinement, showed high reliability and convergent validity, confirming its adequacy within the measurement model. Table 1 presents the detailed results for loadings, AVE, CR, and CA by construct.

**Table 1:** Reliability and convergent validity of constructs.

Construct	First-order construct	Item	Loadings	AVE	CR	CA
Service quality (E-S-QUAL)	Efficiency	In food delivery apps, it is easy to access any section.	0.854	0.774	0.952	0.951
		In food delivery apps, it is easy to find what I need.	0.867			
		In food delivery apps, the information is well organized.	0.903			
		Food delivery apps load quickly.	0.873			
		Food delivery apps allow me to place an order quickly.	0.899			
		Food delivery apps would allow me to make a purchase quickly.	0.877			
		Food delivery apps are well organized.	0.883			
	System availability	Food delivery apps are always available for placing orders.	0.931	0.876	0.861	0.858
		Food delivery apps download and function immediately.	0.940			
	Fulfillment	Food delivery apps deliver orders within the promised time.	0.834	0.680	0.927	0.921
		Food delivery apps make orders available for delivery within a suitable timeframe.	0.855			
		Food delivery apps deliver what I request quickly.	0.832			
		Food delivery apps have in stock the items they offer.	0.817			
		Food delivery apps fulfill their offers.	0.830			
		Food delivery apps do not make errors in orders.	0.746			
		Food delivery apps fulfill their delivery promises.	0.852			
	Privacy	Food delivery apps protect information about my purchase behavior.	0.923	0.747	0.900	0.836
		Food delivery apps do not share my personal information with other apps.	0.760			
		Food delivery apps protect credit card information.	0.901			
Working conditions		Food delivery apps respect workers’ rights.	0.899	0.865	0.970	0.969
		Food delivery apps ensure safe and non-hazardous working conditions.	0.940			
		Food delivery apps provide decent working conditions.	0.947			
		Food delivery apps treat workers fairly.	0.938			
		Food delivery apps develop, support, and train their workers.	0.922			
		Food delivery apps communicate openly and honestly with their workers.	0.935			
Intention to use		When needed, I intend to use food delivery apps.	0.942	0.916	0.959	0.954
		When needed, I believe I will use food delivery apps again.	0.974			
		When needed, I would like to use food delivery apps again.	0.955			
Intention to recommend		If someone asked me about food delivery apps, I would give a positive opinion.	0.957	0.908	0.950	0.949
		If I had the chance, I would highlight the advantages of food delivery apps.	0.948			
		I would recommend food delivery apps.	0.954			
Socially conscious consumption		I consider the ethical reputation of food delivery companies when ordering.	0.904	0.780	0.875	0.860
		I deliberately avoid ordering from food delivery companies with unethical practices.	0.876			
		I deliberately use food delivery services that offer fair working conditions.	0.868			

Discriminant validity was assessed using two complementary criteria: the Fornell–Larcker criterion and the heterotrait–monotrait ratio (HTMT). According to the Fornell–Larcker criterion, the square root of the AVE of each construct should be greater than its correlation with other constructs in the model. As shown in Table 2, this condition was satisfied in all cases (Fornell and Larcker, 1981). Regarding the HTMT criterion (Table 3), the values obtained for all pairs of constructs were below the threshold of 0.90, indicating adequate discriminant validity between latent variables (Henseler et al., 2016; Voorhees et al., 2016).

**Table 2:** Discriminant validity based - Fornell-Larcker criterion.

	Socially conscious consumption	Fulfillment	Efficiency	Intention to recommend	Intention to use	Privacy	System availability	Perceived working conditions
Socially conscious consumption	0.883							
Fulfillment	0.476	0.821						
Efficiency	0.419	0.640	0.880					
Intention to recommend	0.530	0.699	0.630	0.953				
Intention to use	0.414	0.596	0.658	0.716	0.957			
Privacy	0.412	0.572	0.479	0.516	0.545	0.864		
System availability	0.392	0.637	0.738	0.586	0.576	0.501	0.936	
Perceived working conditions	0.396	0.460	0.254	0.454	0.282	0.423	0.283	0.930

**Table 3:** Discriminant validity based – HTMT ratio.

	Socially conscious consumption	Fulfillment	Efficiency	Intention to recommend	Intention to use	Privacy	System availability
Fulfillment	0.525						
Efficiency	0.457	0.675					
Intention to recommend	0.580	0.742	0.661				
Intention to use	0.450	0.625	0.688	0.749			
Privacy	0.472	0.624	0.498	0.548	0.579		
System availability	0.449	0.709	0.817	0.648	0.634	0.558	
Perceived working conditions	0.428	0.485	0.262	0.470	0.291	0.451	0.307

### 4.2 Second-order construct assessment

Service quality was modeled as a reflective higher-order construct (second-order) composed of four first-order dimensions: efficiency, fulfillment, system availability, and privacy. To estimate this hierarchical model, we applied the disjoint two-stage approach in PLS-SEM, whereby in the first stage the latent variable scores of the first-order constructs are obtained, and in the second stage these scores are used as manifest indicators of the second-order construct (Becker et al., 2012; Sarstedt et al., 2019). This procedure is consistent with the general two-step logic of structural equation modeling, which emphasizes establishing measurement quality prior to the assessment of structural relations (Anderson, 1988), and aligns with the literature on multidimensional constructs (Wright et al., 2012).

As shown in Table 4, all dimensions of the second-order construct exhibited loadings above 0.70 and satisfied the required thresholds for Average Variance Extracted (AVE), Composite Reliability (CR), and Cronbach’s alpha (CA), thereby confirming the validity of the hierarchical model. In addition, and consistent with best practice for higher-order models (Sarstedt et al., 2019), we examined collinearity diagnostics among the first-order dimensions. All VIF values ranged between 1.7 and 2.8, which are well below the recommended cut-off of 3.3 (Hair et al., 2022), confirming the absence of multicollinearity issues in the higher-order measurement model. For completeness, we also inspected item-level collinearity for the E-S-QUAL dimensions; all outer VIFs were below 5.0.

**Table 4:** Evaluation of the second-order construct: service quality.

Construct	Items	Loadings	VIF	CA	CR	AVE
Service quality (E-S-QUAL)	Fulfillment	0.834	2.4	0.822	0.826	0.653
	System availability	0.849	1.7			
	Efficiency	0.815	2.8			
	Privacy	0.742	1.9			

Overall, these results indicated that the hierarchical measurement model demonstrates adequate reliability, convergent validity, and discriminant validity, providing a solid foundation for the subsequent evaluation of the structural model.

### 4.3 Structural model assessment

We used bootstrapping with 5,000 resamples, two-tailed tests, and 95% confidence intervals to obtain standard errors, *t*-values, and confidence intervals for all direct and indirect effects. Before analyzing the structural relationships among the constructs, the overall model fit was assessed using the Standardized Root Mean Square Residual (SRMR) index. The model yielded a value of 0.057, indicating a good fit according to the established thresholds for PLS-SEM models (Henseler et al., 2016). For completeness, additional exact-fit indices were also inspected (dULS = 0.612; dG = 0.367), which are in line with values typically reported in prediction-oriented PLS-SEM studies.

To address the potential risk of common-method bias associated with the single-source cross-sectional design, both procedural and statistical remedies were applied. Procedurally, anonymity and confidentiality were ensured, and items were randomized within the online questionnaire to reduce evaluation apprehension and consistency artifacts. Statistically, we conducted the full collinearity assessment test as recommended by Kock (2015). The full collinearity VIFs were 2.89 for service quality, 2.11 for working conditions, 1.76 for socially conscious consumption, 2.54 for intention to use, and 2.42 for intention to recommend. All values were well below the conservative threshold of 3.3, indicating the absence of common-method variance issues and supporting the robustness of the observed relationships.

**Figure 2:**
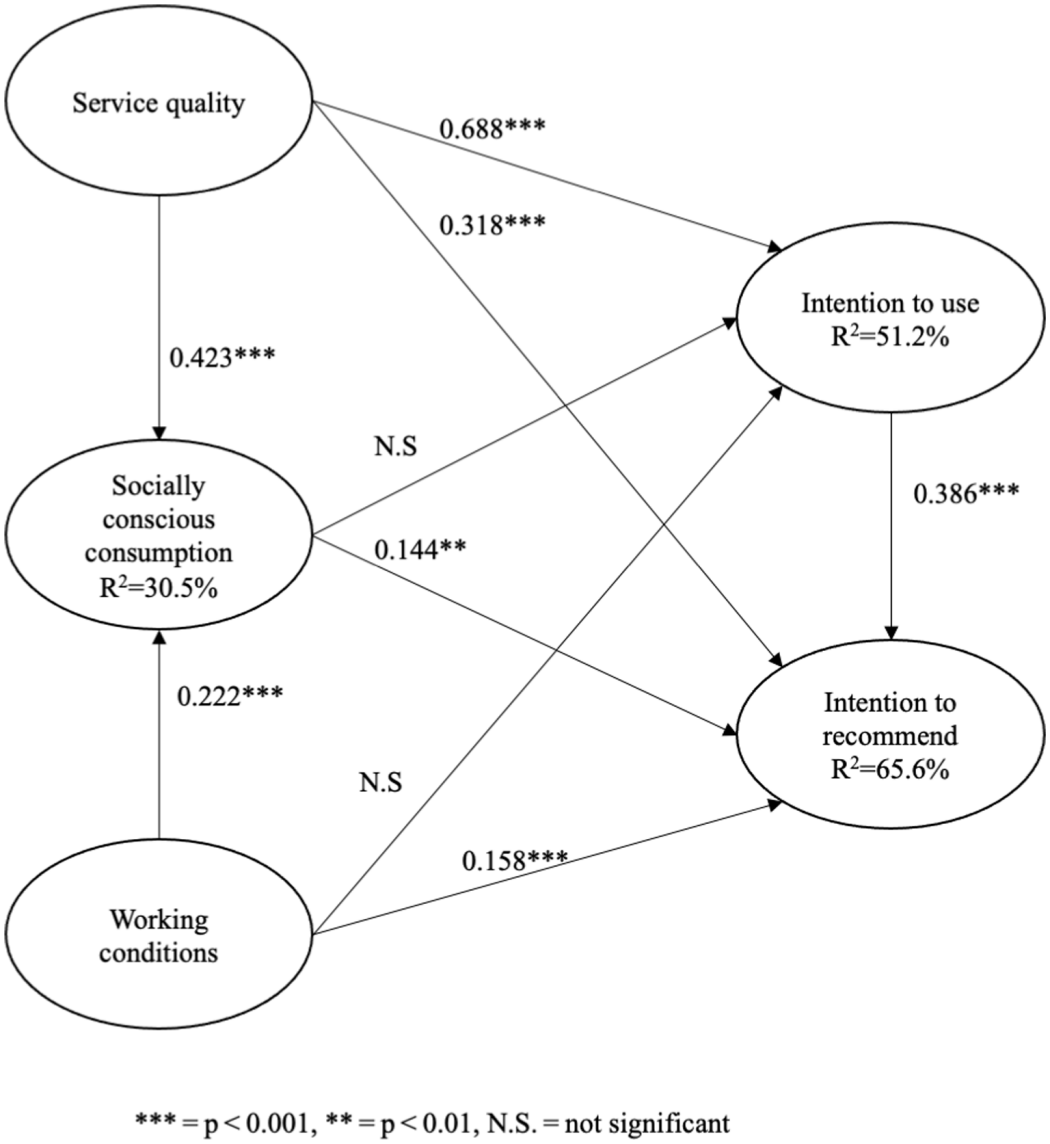
Model results.

The results showed that the stimuli included in the model explained 30.5% of the variance in socially conscious consumption, 51.2% of the variance in intention to use, and 65.6% of the variance in intention to recommend (Figure 2). These values suggest a moderate to high explanatory capacity, particularly with respect to relational consumer behaviors such as service recommendations.

Table 5 reports standardized path coefficients (*β*), *t*-values, two-tailed 95% confidence intervals, and *p*-values obtained via bootstrapping. The hypotheses proposed within the SOBC framework are partially supported. Service quality exerts significant positive effects on socially conscious consumption, intention to use, and intention to recommend, reinforcing the central role of functional attributes (efficiency, system availability, fulfillment, privacy) in shaping both individual and relational behaviors on food delivery platforms. Working conditions significantly affect socially conscious consumption and intention to recommend but not intention to use. Socially conscious consumption significantly predicts intention to recommend but not intention to use. Finally, intention to use significantly predicts intention to recommend.

**Table 5:** Hypotheses testing results.

Hypothesis	Path	*β*	*t*-value	95% CI	*p*-value	Result
H1	Service quality → Socially conscious consumption	0.423	11.27	[0.315, 0.531]	0.000	Supported
H2	Service quality → Intention to use	0.688	21.61	[0.601, 0.769]	0.000	Supported
H3	Service quality → Intention to recommend	0.318	6.84	[0.212, 0.420]	0.000	Supported
H4	Working conditions → Socially conscious consumption	0.222	6.37	[0.150, 0.301]	0.000	Supported
H5	Working conditions → Intention to use	−0.029	0.64	[−0.116, 0.060]	0.522	Not supported
H6	Working conditions → Intention to recommend	0.158	4.18	[0.088, 0.229]	0.000	Supported
H7	Socially conscious Consumption → Intention to use	0.072	1.35	[−0.025, 0.188]	0.179	Not supported
H8	Socially conscious consumption → Intention to recommend	0.144	3.27	[0.066, 0.228]	0.001	Supported
H9	Intention to use → Intention to recommend	0.386	9.45	[0.270, 0.496]	0.000	Supported

In addition to the analysis of path significance, we assessed the effect sizes (f^2^) of each predictor on its endogenous constructs by comparing the explained variance of the full model with models where each predictor was omitted in turn (Cohen, 1988). Table 6 reports the f^2^ values, which indicate the relative contribution of each predictor beyond statistical significance. Following conventional thresholds (0.02 = small, 0.15 = medium, 0.35 = large), values below 0.02 are considered negligible or trivial (Cohen, 1988).

**Table 6:** Effect sizes (f^2^).

Endogenous construct	Predictor	f^2^	Effect size
Socially conscious consumption	Service quality	0.214	Medium
	Working conditions	0.059	Small
Intention to use	Service quality	0.664	Large
	Working conditions	0.001	Trivial
	Socially conscious consumption	0.007	Trivial
Intention to recommend	Service quality	0.115	Small
	Working conditions	0.057	Small
	Socially conscious consumption	0.042	Small
	Intention to use	0.211	Medium

The results show that service quality exerts the strongest influence, with a large effect on Intention to Use (f^2^ = 0.664), a medium effect on socially conscious consumption (f^2^ = 0.214), and a small-to-medium effect on intention to recommend (f^2^ = 0.121). Working conditions have only a small effect on socially conscious consumption (f^2^ = 0.059) and intention to recommend (f^2^ = 0.057), and a negligible effect on intention to use (f^2^ = 0.001). Socially conscious consumption contributes negligible effects on both intention to use (f^2^ = 0.007) and intention to recommend (f^2^ = 0.041). Finally, Intention to use exerts a medium effect on intention to recommend (f^2^ = 0.211), confirming its role in linking functional assessments to relational consequences. Overall, these results reinforce the theoretical expectation that functional quality strongly drives immediate behavioral responses, whereas ethical-social considerations have more limited predictive power, shaping evaluations and downstream recommendations only modestly.

### 4.4 Indirect effects

We tested indirect paths using 5,000 bootstrap resamples (two-tailed 95% CIs). Results in Table 7 show that service quality has a significant total indirect effect on recommend (*β* = 0.338, *p* < 0.001), decomposed mainly via use [Service quality → Use → Recommend: *β* = 0.265, 95% CI (0.187, 0.341)] and, to a lesser extent, via SCC [Service quality → SCC → Recommend: *β* = 0.061, 95% CI (0.025, 0.099)]. The serial route Service quality → SCC → Use → Recommend is positive but not significant [*β* = 0.012, 95% CI (−0.005, 0.032)].

**Table 7:** Indirect effects.

Indirect path	*β*	*t*-value	95% CI	Result
Service quality → Intention to use → Intention to recommend	0.265	6.807	[0.187, 0.341]	Supported
Service quality → Socially conscious consumption → Intention to recommend	0.061	3.178	[0.025, 0.099]	Supported
Service quality → Socially conscious consumption → Intention to use → Intention to recommend	0.012	1.254	[−0.005, 0.032]	Not Supported
Working conditions → Socially conscious consumption → Intention to recommend	0.032	2.627	[0.011, 0.058]	Supported
Working conditions → Intention to use → Intention to recommend	−0.011	0.646	[−0.045, 0.023]	Not Supported
Socially conscious consumption → Intention to use → Intention to recommend	0.028	1.245	[−0.011, 0.076]	Not Supported

For working conditions, the specific indirect effect via SCC on recommend is significant [working conditions → SCC → Recommend: *β* = 0.032, 95% CI (0.011, 0.058)], whereas the total indirect effect is small and not significant (*β* = 0.027, 95% CI crosses zero) due to a negligible indirect component through Use. The indirect effect SCC → Use → Recommend is not significant (*β* = 0.028, 95% CI includes 0). These results align with the SOBC mapping that emphasizes Use as a proximal behavior (B) and recommend as a downstream consequence (C), with SCC operating as a value-laden cognitive mechanism (O) that channels ethical-social appraisals into relational consequences.

Taken together, these findings indicate that service quality exerts both direct and indirect effects on recommendation, with indirect channels accounting for a substantial share of the total effect, mainly via Use and secondarily via SCC. In contrast, working conditions influence recommendation primarily through SCC, underscoring that ethical appraisals translate into symbolic, relational consequences rather than transactional use. These results reinforce the theoretical mapping of the SOBC model and provide empirical evidence for the coexistence of sequential and shortcut paths in platform-based consumption.

### 4.5 Predictive relevance and out-of-sample prediction

To further evaluate the model’s predictive capability, we assessed predictive relevance (Q^2^) using the blindfolding procedure (omission distance = 7) and applied the PLSpredict procedure (Shmueli et al., 2019). Table 8 presents the Q^2^ values for the three endogenous constructs. All Q^2^ values were greater than zero, confirming the model’s predictive relevance (Chin, 2010; Hair et al., 2019).

**Table 8:** Predictive relevance (Q^2^).

Endogenous construct	Q^2^	Interpretation
Socially conscious consumption	0.296	Medium
Intention to use	0.501	Large
Intention to recommend	0.556	Large

The results indicate that the model exhibits medium predictive relevance for Socially Conscious Consumption and strong predictive relevance for both Intention to Use and Intention to Recommend, underscoring the robustness of the model in predicting key behavioral outcomes.

In addition, PLSpredict was performed to assess out-of-sample prediction accuracy. Table 9 compares the prediction errors (RMSE and MAE) of the PLS model against a linear regression benchmark (LM).

**Table 9:** PLSpredict results (comparison of PLS vs. LM).

Construct	RMSE (PLS)	MAE (PLS)	RMSE (LM)	MAE (LM)	Avg. Loss Diff.	p-value
Socially conscious consumption	0.844	0.649	0.931	0.703	−0.056	0.022
Intention to use	0.711	0.500	0.698	0.497	+0.003	0.905
Intention to recommend	0.670	0.486	0.672	0.491	−0.006	0.806
Overall					−0.020	0.246

The PLSpredict results confirm that the model achieves lower prediction errors than the linear benchmark for socially conscious consumption, with significant improvements in predictive accuracy (*p* = 0.022). For intention to use and intention to recommend, PLS and LM perform similarly, which is consistent with the strong explanatory and predictive power already indicated by R^2^ and Q^2^. Taken together, these findings strengthen the empirical robustness of the model and provide additional support for its practical implications in digital platform contexts.

## 5 Discussion

The results confirm the central role of service quality as a determinant of consumer behavior in food-delivery applications. This construct exhibits a strong effect on intention to use (*β* = 0.688, large f^2^) and a more moderate direct effect on intention to recommend (*β* = 0.318), accompanied by a substantive indirect effect via intention to use (*β* = 0.265). These findings reinforce prior evidence that attributes such as efficiency, fulfillment, system availability, and privacy anchor functional evaluations of service performance and are associated with loyalty and continuance in digital environments (Fan et al., 2022; Vu et al., 2023; Yeo et al., 2017). A one-standard-deviation improvement in service quality is associated with approximately a 0.64-standard-deviation increase in recommendation intention. Among its dimensions, system availability shows the strongest contribution to the higher-order construct (loading = 0.849; Table 4), suggesting that reliability and technical stability are promising levers for managerial action.

Second, service quality also activates internal socially conscious consumption (SCC) processes. This relationship suggests that a service perceived as reliable and secure can create room for more favorable evaluations of a firm’s ethical and social practices (Ali et al., 2023; Erkmen and Turegun, 2022). Within the SOBC framework, this indicates that functional stimuli not only trigger immediate transactional behaviors but can also facilitate value-laden evaluations that manifest in relational consequences such as recommendation.

Regarding perceived working conditions, the results show significant effects on the organism (SCC) and the consequence (intention to recommend), but not on the transactional behavior (intention to use). This asymmetry aligns with studies showing that, although socially conscious consumers penalize platforms that fail to meet basic labor standards, such penalties manifest more often in symbolic behaviors (e.g., recommending or not) rather than in service abandonment (Belanche et al., 2021; Goods et al., 2024). Evidence also shows that brand-related perceptions act as mediating organisms that filter quality evaluations into satisfaction and behavioral responses, reinforcing the idea that consumers often process functional and symbolic dimensions differently (Erkmen and Turegun, 2022). This pattern suggests that ethical appraisals operate mainly through reputational and value-expression mechanisms, whereby consumers signal their values by recommending or withholding recommendation rather than altering their own usage.

Consistently, SCC influenced recommendation (*β* = 0.144) but not intention to use. This pattern supports the idea that ethical motivations are expressed more in relational and reputational spheres than in immediate consumption choices (Kim et al., 2023; Vu et al., 2023). Recommendation thus emerges as a symbolic act of value expression, consistent with the SOBC distinction between behavior (B) and consequence (C).

Analyses of indirect effects and effect sizes add nuance. Service quality exerts both direct and indirect effects on recommendation, with the sequential path through use being most influential. By contrast, working conditions affect recommendation primarily through the organism, confirming that ethical concerns are cognitively and symbolically channeled before becoming visible consequences. These results are supported by the model’s predictive relevance (positive Q^2^ and PLSpredict gains for SCC), reinforcing its explanatory capacity and practical applicability (Hair et al., 2019).

At the same time, behavioral outcomes associated with the use of these applications are not exclusively positive. Alongside convenience and satisfaction, recent research shows that choice architecture and digital nudging can amplify overconsumption (Jesse et al., 2021; Lohmann et al., 2024), that compulsive use of delivery apps is associated with less healthy dietary patterns (Singh et al., 2024), and that incentives and ease of use encourage overordering and food waste (Shankar et al., 2022). These dynamics define a dual-valence outcome space in which the same technological affordances that enhance convenience and satisfaction may also entail public-health and sustainability costs.

Taken together, the findings confirm the utility of the SOBC model for capturing the interplay between functional and ethical–social stimuli, evaluative processes, immediate behaviors, and relational consequences. They also reveal the structural limitations of responsible consumption: social consciousness exerts a stronger influence on symbolic behaviors (e.g., reputation and recommendation) than on immediate usage decisions, suggesting that consumer action should be complemented by regulatory frameworks and structural policies to ensure fair working conditions (Goods et al., 2024; Kim et al., 2023).

Finally, recent evidence broadens our reading of dual outcomes in food delivery apps. On one hand, advances in algorithmic functionalities (e.g., personalization) can strengthen task–technology fit and emotional trust, promoting adoption; yet they also introduce privacy and bias risks that delimit the translation from B to C (Chakraborty, 2025). On the other hand, from the perspective of the commercial determinants of health, platforms tend to intensify the availability and promotion of less healthy options while sustaining precarious labor schemes, with population-level and occupational implications (Bennett et al., 2025; Benson et al., 2025). Collectively, these findings underscore the need for SOBC models that incorporate algorithmic stimuli and negative mediators/moderators (e.g., perceived risk, waste propensity) (Jabbour Al Maalouf et al., 2025; Seo and Roh, 2025), and e-WOM loops through which these evaluations diffuse (Boldureanu et al., 2025). Moreover, environmental concerns are emerging as levers of intention and use among younger segments, pointing to “green” logistics and packaging strategies with reputational, and increasingly, behavioral returns (Chantasoon et al., 2025).

## 6 Implications

The findings of this study contribute to the literature on consumer behavior on digital platforms by validating the SOBC model in the context of food delivery applications, incorporating both functional and ethical-social dimensions as stimuli. In particular, the integration of socially conscious consumption as the organism, that is, the cognitive–affective mechanism between stimuli and behaviors, reinforces emerging lines of research on ethical and responsible consumption in digital environments (Belanche et al., 2021). Moreover, the pattern whereby labor-practice perceptions shape recommendation and SCC more than use underscores the role of reputational and value-expression mechanisms in platform contexts.

From a theoretical standpoint, this study underscores the need to further understand the conditions under which perceptions of labor practices affect consumer decision-making. Previous research indicates that the effects of these perceptions may vary depending on consumers’ levels of social consciousness, suggesting the relevance of including moderating variables, such as prosocial orientation or moral identity, in future models (Belanche et al., 2021; Goods et al., 2024). Future theorizing should explicitly model these reputational/value-expression channels (e.g., identity signaling) alongside utilitarian pathways to usage.

Moreover, the limited direct influence of social consciousness on usage behavior, as opposed to its stronger impact on recommendations, raises important questions about the gap between beliefs and actions in convenience-driven contexts. This ethical paradox should be explored through mixed-methods approaches and longitudinal designs that allow researchers to observe the evolution of consumers’ moral judgment toward platform-mediated services as well as to examine potential differences across countries or geographic regions. Experimental and panel designs could test when symbolic sanctions (recommendation withholding) translate into behavioral change.

From a practical perspective, the results confirm that service quality remains the most decisive stimulus for encouraging usage behavior, reaffirming the relevance of the E-S-QUAL model in delivery service contexts. Managers should, therefore, focus their efforts on ensuring efficiency, system availability, promise fulfillment, and data protection, thereby delivering a seamless and trustworthy digital experience (Parasuraman et al., 2005; Yeo et al., 2017).

Regarding working conditions, although their direct impact on purchasing behavior is limited, their influence through the organism, and consequently, is significant. This suggests that improving labor conditions may serve as an effective differentiation strategy to attract socially conscious consumers, especially in saturated markets, where customer retention is critical (Belanche et al., 2021; Goods et al., 2024). Because the impact is primarily reputational, firms should complement substantive improvements with credible, verifiable communication (e.g., third-party audits, transparency dashboards) to activate SCC and recommendation.

Companies should actively communicate their ethical commitments and labor conditions as part of their value proposition, as this may activate consumers’ coalitional power to legitimize more sustainable business models (Goods et al., 2024). Additionally, firms may explore the implementation of dual employment models, combining permanent employees with occasional collaborators, to maintain operational flexibility without sacrificing labor fairness. In summary, food delivery platforms may transform social responsibility into a competitive advantage if they integrate ethical improvements into the functional experience, which may lead to greater positive outcomes, such as recommendation, favorable reputation, and customer retention, especially among socially conscious segments.

## 7 Conclusion

The Stimulus–Organism–Behavior–Consequence (S–O–B–C) model was used as the theoretical framework to examine how perceptions of service quality, perceived working conditions, and socially conscious consumption influence intention to use and recommend food delivery applications. The empirical findings demonstrate that service quality is the most decisive stimulus, that significantly affects both immediate behavior (intention to use) and the subsequent consequence (intention to recommend), while also activating moral evaluative processes.

Moreover, the results confirm that perceived working conditions, although not directly influencing usage behavior, have a positive impact on both the organism (socially conscious consumption) and consequence (intention to recommend). This suggests that labor justice functions more as a symbolic and reputational stimulus than a transactional one, and that the evaluative organism primarily operates in the realm of social validation. Accordingly, reputational and value-expression mechanisms help explain why ethical appraisals surface more strongly in recommendation than in immediate use.

This study validates the usefulness of the SOBC model as a robust analytical framework for integrating functional, ethical, behavioral, and relational dimensions in the analysis of digital consumer behavior. At the same time, it highlights a key limitation of ethical consumption: the gap between consumers’ moral judgment and their actual usage behavior, particularly in contexts where convenience-driven logic predominates. Bridging this gap likely requires both operational excellence and credible labor-practice improvements that can move consumers beyond symbolic endorsement.

In a landscape where digital platforms face increasing demands for social responsibility, these findings provide valuable evidence for both theoretical advancement in ethical consumption literature and strategic decision-making in digital service management. Promoting an environment that combines operational efficiency with fair labor practices not only strengthens consumer relationships but also contributes to building a more equitable and sustainable digital economy.

This study is not without limitations. First, the cross-sectional and self-reported design constrains causal inference and may introduce common method bias, despite the procedural remedies applied during data collection. Second, the sample was drawn from a single national context, which restricts the generalizability of the findings across diverse cultural and market settings. Third, the construct of system availability was measured with only two items after indicator refinement; although it showed high reliability and convergent validity, abbreviated scales may reduce stability over time and should be interpreted with caution. Finally, the intercept-based, non-probability sampling strategy limits external validity. The findings are most applicable to digitally active, urban consumers in comparable markets and should be understood as analytical rather than statistical generalization. While the use of demographic and geographic quotas based on national statistics helped mitigate selection biases, the design may under-represent less mobile populations. Additionally, the privacy construct reflects users’ perceptions of data protection and non-sharing, which respondents cannot directly verify. This epistemic limitation is acknowledged as a boundary of perception-based measures.

Future research could address these limitations by employing longitudinal or experimental designs, testing the model in cross-country settings, and validating system availability with the full E-S-QUAL scale. Moreover, the present study focused exclusively on positive behavioral outcomes. Recent evidence suggests that food delivery apps may also foster problematic dynamics, such as compulsive use or overordering that contributes to food waste, with important public health and sustainability implications (Goods et al., 2024; Pepper et al., 2010). Incorporating these negative outcomes into the SOBC framework—as competing mediators or moderators alongside socially conscious consumption—would provide a more comprehensive view of digital consumption. Such an extension would help explain how service quality and working conditions not only encourage engagement and recommendation but may also coexist with, or even exacerbate, harmful patterns of use. Extending the SOBC architecture with algorithmic stimuli and negative outcome pathways (e.g., waste propensity, problematic use) can clarify when symbolic responses become behavioral change, and when they do not.

## Data Availability

The original contributions presented in the study are included in the article/supplementary material, further inquiries can be directed to the corresponding author.
